# Comparative Assessment of the Stress Response of Cattle to Common Dairy Management Practices

**DOI:** 10.3390/ani13132115

**Published:** 2023-06-26

**Authors:** Katja Koenneker, Martin Schulze, Laura Pieper, Markus Jung, Marion Schmicke, Fritz Beyer

**Affiliations:** 1 Institute for Reproduction of Farm Animals Schönow, 16321 Bernau, Germany; m.schulze@ifn-schoenow.de (M.S.); l.pieper@ifn-schoenow.de (L.P.); m.jung@ifn-schoenow.de (M.J.); 2 Clinic for Cattle, Veterinary Endocrinology and Laboratory Diagnostic, University of Veterinary Medicine Hannover, 30559 Hannover, Germany; marion.schmicke@tiho-hannover.de

**Keywords:** dairy cattle, stress, welfare, dairy management, serum cortisol, hypothalamic-pituitary-adrenal axis

## Abstract

**Simple Summary:**

The maintenance of good animal welfare through the implementation of low-stress animal husbandry practices is in the best interest of both the producers of animal products and consumers alike. Previous studies examining acute and chronic stressors in dairy cattle have not provided a scientific basis for assessing how the management practices regularly implemented on commercial dairy farms are perceived by cattle. The aim of the present study was to compare the impact of routine stimuli: milking, veterinary examination, different breeding methods, and hoof trimming on stress hormones and milk production to identify factors that may lead to a stress reaction. The identification of these factors will allow farmers to make more informed management choices to improve not only the welfare but also the health and productivity of cattle within dairy herds.

**Abstract:**

While studies have been conducted examining the stress response of dairy cattle to individual acute and chronic stressors, the results are difficult to compare due to differences in study design and analysis methods. The aim of the present study was to conduct a comparative assessment of the impact of eight common stimuli: artificial insemination (AI), embryo transfer (ET), morning milking (MM), evening milking (EM), veterinary examination (VE), ultrasound examination (US), hoof trimming (HT), and natural breeding (NB) on the hypothalamic-pituitary-adrenal axis and milk production of 24 Holstein-Friesian cattle. After random allocation into control and treatment groups, a total of five blood samples were collected 40 min (Ba1) and 20 min (Ba2) prior to stimulus application, immediately following the stimulus (St), as well as 20 min (Re1) and 40 min (Re2) post-stimulus. A comparison between the overall serum cortisol concentrations in the treatment groups showed a significant difference between HT to AI (*p* = 0.006), ET (*p* = 0.010), MM (*p* = 0.021), VE (*p* = 0.009), EM (*p* = 0.007), and US (*p* = 0.010), except for NB (*p* = 0.542). There is no significant difference between the control groups (*p* > 0.05). The stimuli HT (*p* < 0.001) and NB (*p* < 0.001) showed significant increases in cortisol following stimulus application, and the levels failed to decrease significantly by sample Re2. No significant differences in daily milk yield (kg) were measured amongst the tested stimuli (*p* = 0.472) nor amongst the groups ‘Control’, ‘Treatment’ and ‘no stimulus’ (*p* = 0.350). In conclusion, when factors such as increased physical activity, novel social interaction, sexual arousal, and a more intense restriction of movement are present, the animal’s perceived controllability and predictability might decrease, affecting the animal’s response to stress. Treatments carried out while animals are restrained in a headlock while remaining within their regular group likely result in a less intense activation of the HPA axis.

## 1. Introduction

The impact of farming on environmental sustainability and animal health and welfare has become an increasingly important topic amongst the public in recent years. The concepts of stress and welfare are highly emotive, and in order to avoid anthropomorphic interpretations, the discussion must be based on quantifiable and comparable scientific data. 

Animal welfare refers to the state of an animal as it copes with the environment in which it lives [1]. When evaluating the welfare of an animal, three main questions must be considered: how is the animal functioning (e.g., health and productivity status)? How is the animal feeling (absence of pain, fear, hunger, etc.)? and is the animal able to express natural behavior [2]? Ensuring good animal welfare on dairy farms requires the consideration of non-animal-based and animal-based factors such as housing, handling, social interactions, body condition, the prevalence of lameness and mortality, etc. Stimuli in the environment that have a negative impact on welfare must be identified and reduced.

One of the earliest definitions of stress was proposed by Selye [3]: “Stress is the nonspecific response of the body to any demand made upon it”. It is important to note that both rewarding as well as aversive situations require a metabolic response to facilitate a reaction. Koolhaas et al. [4] have made the distinction between a ‘response’ and a ‘stress response’ clear by stating that the terms ‘stress’ and ‘stressor’ should be used exclusively to describe stimuli that exceed the ability of an individual to maintain allostasis and in which controllability and/or predictability are not perceived. Accordingly, it is the time it takes for a parameter to return to basal values rather than the immediate size of the base-line discrepancy that is the crucial factor when differentiating between a positive and negative stimulus. Repeated exposure to a stimulus or a stressor can lead to increased predictability (presence of an anticipatory reaction and decreased initial reaction) and controllability (increased speed of recovery and changes in released metabolites), and is known as adaption [5,6]. This is an important evolutionary mechanism, as it prevents organisms from responding unnecessarily to the many harmless stimuli present in their environments, thus conserving energy [7].

Traditionally, the study of stress in cattle has been conducted by measuring hypothalamic-pituitary-adrenal (HPA) axis activity [8]. The presence of real or perceived challenges results in the activation of the hypothalamus and the release of corticotropin-releasing hormone (CRH) and arginine vasopressin (AVP) into the hypothalamic-hypophysial portal system [9]. This triggers the release of adrenocorticotropic hormone (ACTH) from the anterior pituitary gland, which, in turn, controls the production of cortisol within the adrenal cortex [10]. The magnitude and duration of HPA axis activation are regulated by glucocorticoid negative feedback mechanisms at the level of the hypothalamus and pituitary gland [11,12]. Cortisol can be reliably detected in blood, saliva, hair, urine, feces, lacrimal fluid, and even seminal plasma [10,13,14]. It demonstrates ultradian, circadian, and stimulus-induced activity [8,15,16]. Cortisol binds to intracellular mineralocorticoid- [17] or glucocorticoid [18] receptors, which either activate or inhibit gene expression and cellular function [9]. The release of cortisol initially acts to facilitate the fight or flight response in an effort to maintain homeostasis, for example, through the mobilization of energy (the release of insulin and gluconeogenesis), the inhibition of humoral and cell-mediated immunity, increased vigilance, and enhanced cognition [19,20]. Chronic activation can, however, result in homeostatic overload, leading to detrimental effects on health (increased disease susceptibility), productivity (reduced milk yield and growth), and the reproductive capabilities of the organism [21,22].

In addition to the activation of neuroendocrine systems regulated by the HPA axis, cattle respond to stress through the neural activation of the autonomic nervous system (ANS) and, thereby, the sympathetic-adrenal-medullary (SAM) axis [23]. In response to an acute stressor, the activation of the sympathetic nervous system leads to the release of catecholamines (adrenaline and noradrenaline) from the adrenal medulla. These catecholamines circulate throughout the body, interacting with α-adrenergic and β-adrenergic receptors expressed in many organs. This leads to increased blood flow to muscles and the brain, bronchiolar dilatation, gluconeogenesis, reduced intestinal motility, increased arterial pressure, and increased heart rate and cardiac output, as well as enhanced cognition and alertness [24,25]. The activation of the ANS can be effectively measured through changes in heart rate variability (HRV) [26]. This can be accomplished by measuring the variations in time between successive heart beats, also known as the interbeat interval (IBI), using portable heart rate monitors [23,26]. Measuring HRV is superior to only measuring HR, as an increased heartbeat can be the result of increased sympathetic activity, decreased parasympathetic activity, or changes to both systems at once [27]. Only HRV parameters are able to reflect this complex relationship, and their non-invasive nature makes them an ideal choice for farm animals, including cattle.

In addition to the evaluation of stress through the direct activation of the HPA and SAM axis, exposure to stimuli considered stressful by cattle can be reflected indirectly in short- and long-term changes in milk production. The amount of milk secreted by the mammary gland is controlled by diverse biological, hormonal, and environmental factors and is highly sensitive to the effects of stress and illness [2,28]. For example, an acute stressor, such as a novel environment, can lead to the disturbed release of oxytocin from the pituitary gland and, thereby, to a decrease in daily milk yield [29]. Furthermore, nutritional factors affecting muscularity and body condition score have also been shown to have an impact on milk yield and production over time [30].

Improving the well-being of farm animals through the optimization of housing systems and management practices does not only conform with the wishes of modern consumers but is also in the best interest of farmers, as good animal welfare often correlates with improved productivity. Across species, even small changes in the environment, such as removing partitions in aviaries and thus increasing the freedom of movement of laying hens, have been shown to improve animal welfare and species-specific behavior [31]. While a multitude of studies have been conducted in cattle examining physiological and behavioral changes as a result of acute and chronic stressors (for example, [32,33,34]), a lack of standard practices in experimental design and analysis methods has meant that the results are difficult to compare [35]. The main objective of this study was to examine and compare the impact of routine stimuli used in commercial dairy management on the HPA axis, SAM axis, and milk production. This information allows for the evaluation of how well cattle have adapted to their regular environment and the identification of factors that may lead to a stress reaction. These factors can be used to create recommendations for husbandry standards, which can help improve welfare and, in turn, the health and productivity of dairy cattle.

## 2. Materials and Methods

### 2.1. Animals and Housing

For this study, clinically healthy Holstein-Friesian cattle (*n* = 24) were selected from a herd of a conventional dairy farm (*N* ≈ 700) in Brandenburg, Germany (13°28′ E, 52°48′ N). Cows were between 3–4 years of age and at the beginning of their first or second lactation (83 ± 43 days in milk, milk yield avg. 38 kg/day in the 2 weeks prior to the experiment, body condition score 3–4). The experimental group was kept separate from the other animals on the property and was allowed 14 days for adaptation to the new group dynamic. The freestall had a slatted floor, raised cubicles with rubber mats, and an adjoining feeding table outfitted with self-locking headlocks. Animals were accustomed to being restrained in the headlocks while standing whenever check-ups, artificial inseminations, medical treatments, etc., were to be performed. The total mixed ration was fed twice per day at 5:00 a.m. and at 1:00 p.m., and animals had access to water *ad libitum*. The experimental group was milked twice per day at 7:00 a.m. and 7:00 p.m. in a 36-stall rotary milking parlor (Dairyrotor T8900, Co. GEA Group, Düsseldorf, Germany). The remaining 12 places in the carousel were left vacant to avoid any mix-ups. Health status and production data were monitored by the software HERDEplus^®^ (DSP Agrosoft GmbH, Ketzin/Havel, Germany). This project was approved by the State Office for Occupational Safety, Consumer Protection, and Health in Brandenburg (animal welfare number: 2347-37-2020).

### 2.2. Experimental Design

**Figure 1:**
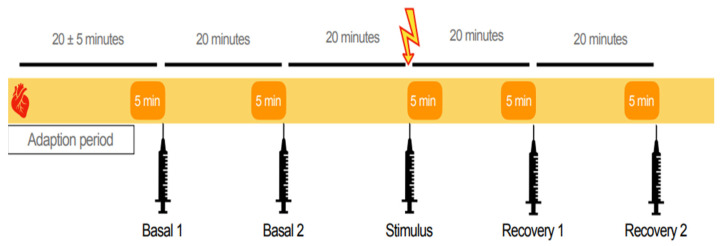
General experimental procedure: in total, five blood samples were collected from all animals (*n* = 24), irrespective of Co or Tr group. Basal blood samples were collected 40 min (Basal 1) and 20 min (Basal 2) prior to stimulus application. The third blood sample (Stimulus) was taken 1–3 min following stimulus application. Finally, the samples were collected 20 (Recovery 1) and 40 (Recovery 2) min after stimulus application to map the possible return of the SCC to baseline. The solid yellow bar represents the continuous recording of HRV parameters from a sub-group of Tr animals (*n* = 5). Analysis of HRV parameters occurred later, using 5 min time windows (orange boxes) [38], and was oriented around the time of blood sample collection.

The stimuli tested in this experiment are routinely performed on commercial dairy farms: artificial insemination (AI), embryo transfer (ET), morning milking (MM), evening milking (EM), veterinary examination (VE), ultrasound examination (US), hoof trimming (HT), and natural breeding (NB). Each stimulus was tested once between February and May 2021, except for NB, which was repeated in May and again in October 2021 due to the failure of the bull to mount the cows. On an experimental day, only one stimulus was tested with 7 ± 6 days as a resting period between experiments, in which the cattle were not sampled or subject to any further examinations. Sampling occurred following morning milking between 10:00 a.m. and 2 p.m., except for the stimulus MM (7:00 a.m.–12:00 p.m.) and EM (6:30–9:30 p.m.). Once the animals were restrained in the headlocks, the experimental group was randomly divided into treatment (Tr) and control (Co) animals. Animals were assigned to the Co group for some stimuli and the Tr group for others. Due to logistical constraints, the division of animals into the Tr and Co groups was not constant across the stimuli. For more detailed information on the individual stimuli and group sizes, refer to Appendix A. The stimulation of the sympathetic-adrenal-medullary (SAM) axis was examined through changes in heart rate variability (HRV). Accordingly, mobile heart rate monitors (POLAR Equine Belt^®^ H7) were secured to a randomly selected sub-group of five Tr animals per experiment, and HRV data were recorded continuously. Following an adaption period to the HR monitors of 20 ± 5 min, serial blood samples were collected from the coccygeal vein for the evaluation of changes in serum cortisol concentration (SCC) over time. A total of five blood samples were collected from each animal, irrespective of the assigned group [36,37]. First, two blood samples were collected 40 min (Basal 1 = Ba1) and 20 min (Basal 2 = Ba2) before the application of the stimulus to determine the basal values and measure the possible effects of sampling. A third sample (Stimulus = St) was taken shortly after stimulus application to measure the possible effects thereof. Finally, a further two samples were collected 20 min (Recovery 1 = Re1) and 40 min (Recovery 2 = Re2) after stimulus application to identify a possible recovery of cortisol levels. The general procedure is represented in Figure 1.

### 2.3. Serum Cortisol

The blood samples were taken from the coccygeal vein using 20-gauge cannulas (0.90 × 40 mm − 20 G × 1½, Co. B. Braun Melsungen AG, Melsungen, Germany) and collected in a 9 mL serum Monovette^®^ (Luer cone 9 mL, Co. SARSTEDT AG & Co. KG, Nürmbrecht, Germany). Collection occurred according to a specified time plan (see Section 2.2) and was conducted by a minimum of two veterinarians to limit the sampling time. Following collection, the blood samples were stored protected from light at a temperature of 12 ± 2 °C and were then centrifuged at 3000× *g* for 10 min. Next, 500 μL of the separated serum was transferred with a micropipette into a 1.5 mL Eppendorf microtube and stored at −20 °C until further analysis. A total of *N* = 875 cortisol samples were collected. The samples were analyzed in the Endocrinological Laboratory of the University of Veterinary Medicine in Hannover. The SCC was determined using a competitive radioimmunoassay (CORTISOL RIA KIT IM1841, Co. Immunotech, Beckman Coulter, via Demeditec Diagnostics GmbH; Kiel, Germany) according to the manufacturer’s instructions. The analytical range of the test kit given by the manufacturer was between 5.0 and 2000 nM, and the intra-assay coefficient of variation for bovine serum was 5.8%. Laboratory personnel was blinded to the group assignment of the samples.

### 2.4. Daily Milk Yield

The farm on which this experiment was performed monitored the health of their herd using the herd management software HERDEplus. As part of the regular practices on the farm, all animals, including the individuals selected for the experimental group, were fitted with a monitoring device in the form of a collar worn around the neck. The farm employed a twice-a-day milking strategy. The milk yield from each milking was recorded and transmitted automatically to the HERDEplus^®^ system. Milk yield (kg) from the morning and evening milking was combined to obtain the daily milk yield (DMY). The production data from a time frame starting 2 weeks prior to testing until 1 week post-testing was considered. Production data from the time of the repeated NB experiment in October was not included.

### 2.5. Heart Rate Variability

Heart rate data from *n* = 5 Tr animals was recorded continuously using a POLAR Equine mobile recording system. This included a belt (Equine Belt^®^ H7, Co. Polar Electro Europe AG, Kempele, Finnland), as well as a sensor with Bluetooth transmission capabilities (H7 Heart Rate Sensor, Co. Polar Electro Europe AG, Kempele, Finnland) containing two electrodes. The electrodes were positioned caudal to the left shoulder blade at elbow height. To minimize electrical resistance and thereby optimize conductivity, the area was shaved, and ample amounts of ultrasound gel were applied. The HR data was collected using a sampling frequency of 1000 Hz and saved within the ELITE HRV-App^®^ (Elite HRV Nr. 5.5.4 Co. ELITE HRV LLC; Asheville, NC, USA), which was installed on a smartphone attached to the sensor belt. Data from the smartphone was later transferred to a computer and analyzed using Kubios HRV^®^ software (Version 3.1.0, Biomedical Analysis and Medical Imaging Group, Department of Applied Physics, University of Eastern Finland, Kuopio, Finland). Following visual inspection, an automatic noise detection filter set to ‘default = medium’ was applied. All segments marked as noise were excluded. The remaining data sets were examined using an automatic artifact correction algorithm, and sections with measurement error rates exceeding 5% were excluded. Data were detrended (smoothness prior’s method), and spectral analysis was performed using fast Fourier transformation (FFT). The frequency ranges were adapted for mature cattle: high frequency (HF) power 0.2 to 0.58 Hz, low frequency (LF) power 0.05 to 0.20 Hz [26]. These frequency ranges have been applied in previous HRV research in dairy cattle [39,40,41]. For each recording, five periods, each with a duration of 5 min, were examined [38]. The periods were selected in similar intervals to the cortisol data. The analyzed HRV parameters were: mean heart rate (Mean HR), mean R-R interval (Mean RR), root mean square of successive differences (RMSSD), high-frequency power (HF power), and the standard deviation of the Poincaré plot perpendicular to and along the line-of-identity (SD2/SD1 ratio).

### 2.6. Statistical Analysis

The statistical evaluation for all data was performed using IBM^®^ SPSS statistics (version 26, Co. SPSS GmbH Software, Munich, Germany). Data processing for the graphical representation of the cortisol data was also conducted using R [42], which was extended by the packages lmerTest [43] for linear mixed models and ggplot2 [44].

The effects of the different stimuli on SCC were analyzed using a general linear mixed model (GLMM). The model included log-transformed SCC in nmol/L as the dependent variable, and ‘Cow ID’ was included as a random effect (covariance type = variance components). Fixed effects were: ‘daily status’ (Control, Treatment), ‘sample’ (Ba1, Ba2, St, Re1, Re2), ‘stimulus’ (AI, ET, MM, EM, VE, US, HT, NB) and the interaction term ‘daily status × sample × stimulus’.

To assess differences in overall cortisol secretion between the stimuli and between daily status groups, a net area under the curve (net AUC) was calculated using a formula described in previous research [45,46]:

net AUC = Σ [(C_n_ + C_n+1_)/2 × h − baseline],

where C is the cortisol concentration measured at the five different time points, and h is the time in hours between the two C values (h = 1/3). The baseline is the cortisol concentration at the sample time point Ba1.

Heteroscedastic data was analyzed using a Welch ANOVA and Games-Howell Post-hoc Test. Homoscedastic data were analyzed with a one-way ANOVA and Tukey post-hoc test. The distribution of the data was graphically represented using a boxplot. The net AUC was presented in absolute values.

For the analysis of the daily milk yield, all cases in which only one milking per day was registered, or ≤ 1 kg of milk was recorded during a milking, were considered missing data and excluded from further analysis. A GLMM with ‘Milk kg’ as the dependent variable and ‘Cow ID’ as a random effect (covariance type = variance components) was constructed. The model included: ‘month’ (February, March, April, May, June), ‘stimulus’ (none, AI, ET, MM, EM, VE, US, HT, NBI, NBII), ‘status’ (no stimulus, Control, and Treatment), and the interaction term of ‘status × stimulus’ as fixed effects.

The data from the HR recordings were impacted by failure of the sensors to maintain contact with the skin during physical activity, noises from muscle action potentials and transmission failures. This resulted in the collection of incomplete HRV recordings: AI (*n* = 4), ET (*n* = 5), MM (*n* = 4), EM (*n* = 3), VE (*n* = 5), US (*n* = 2), HT (*n* = 3) and NB (*n* = 0). Individual GLMM were created for each of the HRV parameters: Mean HR, Mean RR, RMSSD, HF power, and SD2/SD1 ratio, respectively. The models all included ‘Cow ID’ as a random effect (covariance type = variance components) and the fixed effects: sample (pre-stimulus, stimulus, post-stimulus), stimulus (AI, ET, MM, EM, VE, US, HT), and the interaction term ‘stimulus × sample’.

All Post-hoc comparisons were conducted by applying the Bonferroni correction. A *p*-value of <0.05 was considered significant for all tests.

## 3. Results

### 3.1. Serum Cortisol

A one-way ANOVA conducted on the net AUC values found no significant difference (*p* = 0.795) between cows experiencing social contact with a breeding bull (Not mounted: *n* = 9) and cows that mated (Mounted: *n* = 5) with a breeding bull (see Figure S1). For this reason, the groups were not analyzed separately for any of the analyses.

**Figure 2:**
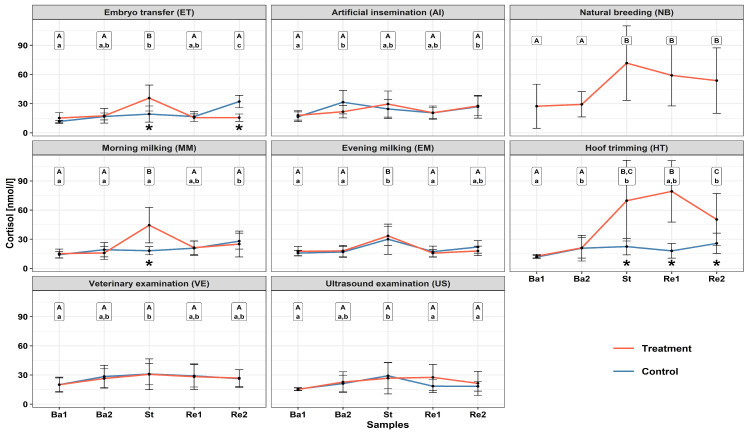
The course of serum cortisol concentrations over time, subdivided for the Co and Tr groups for each stimulus: Ba1 = −40 min before stimulus application, Ba2 = −20 min before stimulus application, St = 0 min, Re1 = +20 min after stimulus application, and Re2 = +40 min after stimulus application. Significant differences between time points within the Co group (^abc^ *p* < 0.05). Significant differences between time points within the Tr group (^ABC^ *p* < 0.05). Significant differences between the Co and Tr groups within each stimulus (* *p* ≤ 0.001). No data from the Co animals were collected during the natural breeding experiment.

**Figure 3:**
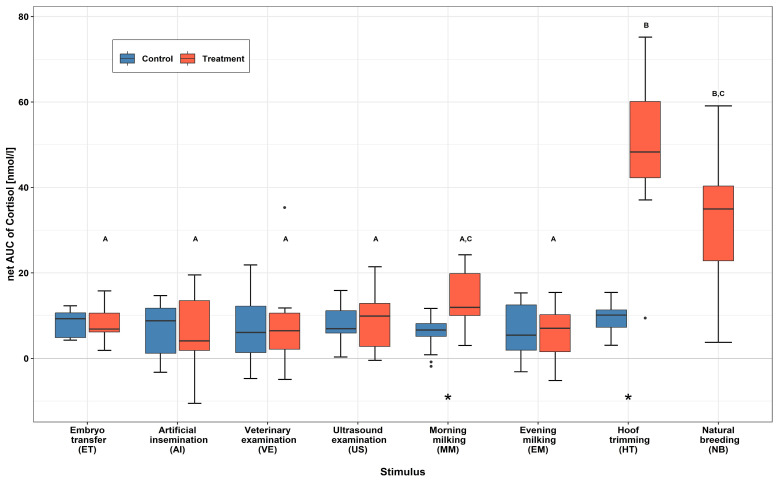
Comparison of overall serum cortisol concentrations. There was no significant difference in overall cortisol concentration measured in net AUC between the Co groups. Significant differences between the Tr groups were identified (^ABC^ *p* < 0.05). * *p* ≤ 0.001 represents significant differences in overall SCC between the Co and Tr groups within each stimulus. No data from the Co animals were collected during the natural breeding experiment. • = values 1.5 times the interquartile range.

The course of SCC over time, subdivided into the control (Co) and treatment groups (Tr) for each stimulus, can be found in Figure 2. Significant increases in SCC were measured within the Tr group directly following stimulus application of the stimuli ET (*p* = 0.001), MM (*p* < 0.001), EM (*p* < 0.001), HT (*p* < 0.001), and NB (*p* < 0.001). The stimuli HT and NB obtained the highest measured individual SCC, 150.8 nmol/L and 140.1 nmol/L, respectively, for sample St. A significant and rapid decrease within 20 min after stimulus application was measured between St and Re1 regarding the stimuli ET (*p* < 0.001), MM (*p* = 0.002), and EM (*p* < 0.001). This was not the case for the stimuli HT (*p* = 0.320) and NB (*p* = 0.557), for which the cortisol levels remained elevated throughout the recovery phase (+40 min). No significant changes in the course of the SCC were measured within the Tr groups for the stimuli AI, VE, or US. The distribution of the net AUC data is represented by boxplots in Figure 3. A significantly higher overall amount of cortisol was secreted following the application of stimulus HT in comparison to AI (*p* = 0.006), ET (*p* = 0.010), MM (*p* = 0.021), EM (*p* = 0.007), VE (*p* = 0.009), and US (*p* = 0.010), with the exception of NB (*p* = 0.542). No significant differences in overall cortisol secretion could be found among the Co groups of the tested stimuli (*p* = 0.558). A significant difference in cortisol secretion between the Co and Tr groups within a stimulus was only found within the treatments MM (*p* = 0.007) and HT (*p* = 0.001). The stimulus NB did not include a Co group due to logistical constraints during testing. The descriptive statistics and estimated mean values from the GLMM can be found in Tables S1 and S2.

### 3.2. Daily Milk Yield

**Figure 4:**
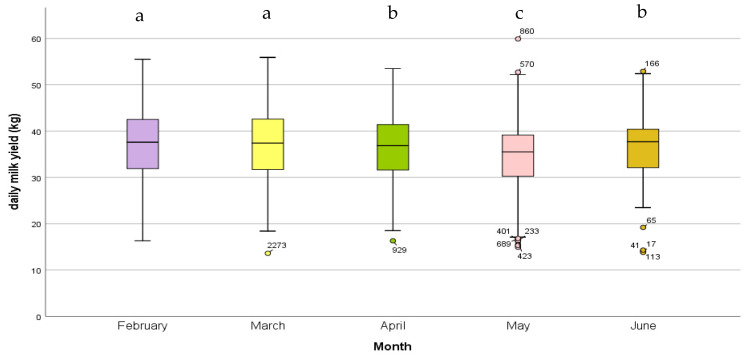
Boxplot representing daily milk yield per month: significant differences between months (^abc^ *p* < 0.05). • = values 1.5 times the interquartile range.

The mean DMY over the entire period was 36.6 ± 1.3 kg (mean ± SE). There was a significant effect for ‘month’ on the DMY of the cows participating in the study (*p* ≤ 0.001), with the month of May delivering the lowest results (34.7 ± 1.3 kg) and the months of February and March showing the highest milk yields (37.5 ± 1.3 kg and 37.4 ± 1.3 kg, respectively). Significant differences in milk yield between months are represented below in Figure 4. There were no significant differences in milk yield among the stimuli (*p* = 0.472). Furthermore, there was no significant differences in the milk yield between the Co and Tr animals within each stimulus (*p* = 0.800). Finally, no significant difference was measured among the status groups ‘Treatment’, ‘Control’, and ‘no stimulus’ (*p* = 0.350).

### 3.3. Heart Rate Variability

The interpretation of the results from the HRV analysis was severely limited, not only by the number of sensors available (*n* = 5) but also by the displacement of the sensor belts, as well as transmission failures. Consequently, the generalizability and significance of the HRV data were seriously reduced, and the influence of the individual stimuli on the SAM axis could not be effectively compared. We would recommend the re-examination of the stimuli using a larger population and equipment optimized to handle the intense physical forces present during the testing of stimuli such as NB. The estimated mean values from the GLMM have been included in Table S3.

## 4. Discussion

In order to gain a better picture of how cattle held on commercial dairy farms perceive their regular environment, eight stimuli commonly encountered by dairy cattle were investigated. The magnitude and duration of the activation of the HPA axis as well as changes in daily milk yield in response to these common stimuli, were compared within one experiment. This meant that the samples were collected and analyzed using common practices, thus mitigating the risks of comparing cortisol analysis results from unrelated studies and different analysis methods [35].

Coccygeal venipuncture using small gauge cannulas was chosen for this study because previous research has indicated that its effect on the HPA axis is minimal, especially in comparison to jugular venipuncture [37,47]. The replicability of the experimental design is demonstrated by the lack of significant differences in overall cortisol secretion (net AUC) amongst the Co groups of the different stimuli, as well as the lack of significant differences between the Co and Tr groups at the Ba1 and Ba2 sampling time points. The SCC measured in the present study, approx. 15–150 nmol/L, is consistent with values previously reported in cattle [48,49].

An overall increased level of cortisol secretion, accompanied by an extended duration of elevated cortisol levels, suggests that the stimuli NB and HT might have been perceived by the animals as less predictable and controllable in comparison to the six other tested stimuli. It is important to recognize, however, that it is not necessarily the stimuli themselves that caused these effects but rather the associated factors. For example, in the NB experiment, no significant difference could be found between the cows that were mounted and those that were not mounted by the bull. This suggests that it was not the act of mating itself that stimulated the HPA axis but the associated factors. As is common practice on conventional dairy farms, the bulls were housed separately from the test herd; this meant that, during the experiment, the cattle were in an unfamiliar environment and experienced novel social interaction. It has been shown that cattle are very sensitive to changes in group dynamics and interactions with animals unfamiliar to them. For instance, a study by von Keyserlingk et al. [50] observed changes in behavior and an acute drop in milk yield when a single cow was introduced to an unfamiliar established group. A further important factor unique to the NB experiment is the sexual arousal experienced by the cattle. Studies completed in stallions [51,52], boars, and bulls [53] have found that sexual arousal significantly increases systemic cortisol concentrations. This is likely in anticipation of the elevated glucose requirements needed for the reproductive act. Although it may be hypothesized that sexual arousal could be perceived as rewarding, its effects on the HPA axis cannot be differentiated from those of the other factors. Considering the negative effects of cortisol on the immunity and the reproductive capacity of cattle, measures that minimize the activation of the HPA axis should be considered.

The results from the HT experiment are comparable to those found by previous studies and are likely related to the intense restriction of movement experienced by cattle when restrained within a hoof-trimming chute [54,55]. A study conducted by Szenci et al. [34] found that heifers restrained in a chute showed a significant increase in plasma cortisol concentration. In comparison, a study by Herskin et al. [56] found no significant increase in cortisol when cattle were restrained for 15 min in a headlock. The intensity of restraint is an important factor that differs between the stimulus HT and stimuli such as AI, ET, VE, and US, where the animals were restrained only in headlocks.

Habituation of the cattle to the stimuli prior to the commencement of the experiment must also be considered. While some studies have reported an increase in cortisol following transrectal palpation [37,57,58], the lack of significant increases in cortisol concentration in the AI and US experiments, as well as the rapid recovery of SCC following ET, suggest that the cattle within this experiment perceived palpation per rectum to be a mild stimulus. Habituation of the lactating dairy cattle may have occurred due to previous exposure during pregnancy checks. This is supported by findings that report differences in reaction following transrectal palpation between lactating and non-lactating mares [59] and cattle [46].

The current study found no significant differences in milk yield among the different stimuli and no differences in milk yield between the Co and Tr animals within each treatment. In addition, no significant differences were found among the groups ‘Control’, ‘Treatment’, or ‘no stimulus’ examined over the entire testing period of 4 months. The fact that daily milk yield remained unchanged on the days an experiment was conducted speaks for the replicability of the experimental design and supports the findings from the cortisol analysis suggesting the ability to control for environmental factors between the different test days. When considering a large number of hormonal, environmental, biological, and nutritional factors that can have an effect on milk yield [28], it is not possible to definitively pinpoint the cause for the change in DMY observed between the months in the present study but it was not likely a result of the conducted experiments. The findings show that exposure to the stimuli did not have a significant short- or long-term impact on milk production and suggests that the cattle perceived the tested stimuli to be mild.

## 5. Conclusions

In summary, the findings from the cortisol analysis suggest that when factors such as increased physical activity, novel social interactions, unfamiliar environments, sexual arousal, and more intense restrictions on movement are present, as was the case during NB and HT, perceived controllability and predictability will likely decrease and a larger corticosterone output will result. Treatments, such as AI, ET, US, and VE that can be carried out when animals are restrained only in a headlock while remaining within their regular group appear to result in the less intense activation of the HPA axis. The habituation of animals to a stimulus must also be considered.

In conclusion, while the present study has highlighted important factors that may help reduce the stress response in dairy cattle to management procedures, further research is required to fully understand the complex relationships and potential long-term effects thereof. The implementation of strategies solely focused on minimizing HPA axis activation may overlook other important aspects of animal welfare and possibly lead to the creation or exploitation of other issues, with negative consequences for the animal and production.

## Data Availability

The data presented in this study are available upon request from the corresponding author.

## Supplementary Materials

The following supporting information is available online at: https://www.mdpi.com/article/10.3390/ani13132115/s1, Figure S1: Stimulus, natural breeding, grouped by animals ‘Mounted’ and ‘Not mounted’ by the bull; Table S1: Descriptive statistics of bovine serum cortisol concentrations arranged by stimulus and subdivided by time point and daily status group; Table S2: Estimated mean bovine serum cortisol concentrations arranged by stimulus and subdivided into Control- and Treatment-groups; Table S3: Estimated mean values of heart rate variability parameters, subdivided by stimulus.

## Appendix A

A common problem identified by Koolhaas et al. [4] in studies focused on stress research is that they are often based on models where naïve animals are exposed to a novel stimulus. They state that while this type of research design may help to determine the physiological limits of a species, it is not a realistic reflection of the regulatory range and adaptive capacity they call upon when modulating their regular environment. The veterinary and farming procedures chosen for further examination in the present study were selected because they represent stimuli that dairy cattle on commercial dairy farms are regularly exposed to. They are listed in the order they were performed.

The first stimulus to be tested was artificial insemination (AI). A fixed-time AI protocol was applied to 16 randomly selected animals to ensure the estrous synchronization of the Tr group. The protocol involved an injection of GnRH (Receptal^®^ 0.004 mg/mL, Co. MSD Tiergesundheit, Rahway, NJ, USA) and the application of an intravaginal progesterone device (CIDR^®^, Co. Zoetis Deutschland GmbH, Berlin, Germany). On day 7 of the protocol, prostaglandin (Estrumate^®^ Co. MSD Tiergesundheit, Rahway, NJ, USA) was injected, and the intravaginal device was removed. The presence of estrous symptoms, such as cervical mucous, swollen labia, and reddened vaginal mucous membranes, was checked by a veterinarian on the day of the experiment. The (*n* = 8) animals with the strongest estrous symptoms were selected to be in the Tr group. The eight remaining stimulated animals were allocated to the Co group (*n* = 15), as they were to be used as recipients for the stimulus ET +7 days later. All the inseminations were performed by the same veterinarian with over 3 years of experience in AI, and the entire procedure took between 2–5 min, from the removal of feces and the cleaning of the outer labia to the intrauterine deposit of semen.

The second stimulus was embryo transfer (ET). The Tr group (*n* = 8) was made up of the stimulated but not inseminated animals from the previous week. The remaining animals (*n* = 16) formed the Co group. An epidural anesthetic with 3.5 mL of procaine (Pronestesic^®^ Co. Veyx-Pharma, Schwarzenborn, Germany) was administered prior to transfer. After passing through the cervix, the device was advanced two-thirds of the way into the uterine horn, ipsilateral to the corpus luteum, at which point the contents of the straw were deposited. Only in half the cases was a viable embryo transferred; in four Tr animals, the ET device was inserted into the uterus without a loaded straw. The time between the administration of the epidural and the transfer of the embryo was approx. 3–6 min.

The experiment’s morning milking (MM) commenced at 7:00 a.m., in comparison to the other stimuli (approx. 10 a.m.). There were *n* = 8 animals in the Tr group and *n* = 15 in the Co group. Once the basal blood samples were taken, the Tr animals walked approx. 100 m to reach the waiting area of the milking parlor. The order in which they entered the milk carousel was not controlled. The Tr group was milked, as per usual, in a 36-stall rotary milking carousel. The remaining 28 places in the carousel were left vacant. The third blood sample (St) was taken from the Tr group while the milking cluster was attached to the udder. Samples from the Co group were taken concurrently by a second veterinarian. The carousel needed 14 min to complete one rotation. The Tr animals walked together back to their stall, and samples Re1 and Re2 were taken from the test herd according to the time plan described under Section 2.2.

The stimulus veterinary examination (VE) was designed to represent the basic general clinical examination performed by veterinarians in the field. It included the examination of the sclera and conjunctiva; the oral examination and measurement of capillary refill time; the auscultation of the heart, lungs, and rumen; the examination of the sub-mandibular, prescapular, and prefemoral lymph nodes, and finally, the measurement of rectal temperature. The exam, including auscultations, took approx. 5 min per animal. There were *n* = 8 animals in the Tr group and *n* = 16 animals in the Co group. The testing day followed the general procedure described under Section 2.2.

The experiment’s evening milking (EM) commenced at 7:00 p.m. There were *n* = 12 animals in the Tr group and *n* = 12 animals in the Co group. The testing day replicated the design for MM described above.

The sixth experiment, ultrasound examination (US), was performed transrectally by an experienced veterinarian using a portable ultrasound console (Easi-Scan, Co. IMV imaging, Bellshill, Scotland, UK) paired with a 7.5 MHz linear transducer and ultrasound goggle-viewer (BUG-VGA™, Co. IMV Imaging, Bellshill, Scotland, UK). There were *n* = 9 animals in the Tr group and *n* = 14 animals in the Co group. The uterine body, uterine horns, and both ovaries were examined (duration approx. 2–3 min), and any pregnancies or abnormal findings were recorded.

The mobile walk-in hoof-trimming (HT) chute was positioned approximately 100 m from the location of the test herd to prevent visual contact and minimize disturbances caused by noise from the machinery (grinders, trimming discs, etc.). After the Ba1 and Ba2 samples were collected, the animals in the Tr group were led to the chute, and hoof trimming was carried out by a professional hoof trimmer familiar with the farm. Only the left hind hoof was trimmed to maximize the number of animals that could be selected for the Tr group while remaining within a reasonable time frame, 6–8 min per animal. The animals were not lame at the time of stimulus exposure did not require any treatment beyond trimming alone. A total of *n* = 8 Tr animals and *n* = 12 Co animals were sampled in the HT experiment according to the general procedure described under Section 2.2.

The animals chosen for the natural breeding (NB) Tr group were in natural heat. The *n* = 5 Tr animals were led as a group to the bullpen approximately 200 m away from the test herd. The Tr animals entered the bullpen individually and were exposed to the bull for 5–8 min. The experiment was considered successful if the bull mounted the cow. The St blood samples were taken after the cow was separated from the bull. Once they were returned to the group, the Re1 and Re2 samples were taken. Due to logistical constraints, it was not possible to sample the Co animals in parallel in this experiment.

The NB experiment was repeated three times due to the failure of the bull to mount the cows in the first two trials, resulting in a total of *n* = 15 samples.

## References

[1] World Organisation for Animal Health (OIE) (2022). Terrestrial Animal Health Code. https://www.woah.org/en/what-we-do/standards/codes-and-manuals/terrestrial-code-online-access/.

[2] von Keyserlingk M.A.G., Rushen J., de Passillé A.M., Weary D.M. (2009). Invited review: The welfare of dairy cattle—Key concepts and the role of science. J. Dairy Sci..

[3] Selye H. (1950). Stress and the general adaptation syndrome. Br. Med. J..

[4] Koolhaas J.M., Bartolomucci A., Buwalda B., de Boer S.F., Flügge G., Korte S.M., Meerlo P., Murison R., Olivier B., Palanza P. (2011). Stress revisited: A critical evaluation of the stress concept. Neurosci. Biobehav. Rev..

[5] de Boer S.F., Koopmans S.J., Slangen J.L., Van Der Gugten J. (1990). Plasma catecholamine, corticosterone and glucose responses to repeated stress in rats: Effect of interstressor interval length. Physiol. Behav..

[6] McEwen B.S. (1998). Stress, adaptation, and disease. Allostasis and allostatic load. Ann. N. Y. Acad. Sci..

[7] Rankin C.H., Abrams T., Barry R.J., Bhatnagar S., Clayton D.F., Colombo J., Coppola G., Geyer M.A., Glanzman D.L., Marsland S. (2009). Habituation revisited: An updated and revised description of the behavioral characteristics of habituation. Neurobiol. Learn. Mem..

[8] Mormède P., Andanson S., Aupérin B., Beerda B., Guémené D., Malmkvist J., Manteca X., Manteuffel G., Prunet P., van Reenen C.G. (2007). Exploration of the hypothalamic–pituitary–adrenal function as a tool to evaluate animal welfare. Physiol. Behav..

[9] Mifsud K.R., Reul J.M.H.M. (2018). Mineralocorticoid and glucocorticoid receptor-mediated control of genomic responses to stress in the brain. Stress.

[10] Spencer R., Deak T. (2017). A users guide to HPA axis research. Physiol. Behav..

[11] Harvey S., Phillips J.G., Rees A., Hall T.R. (1984). Stress and adrenal function. J. Exp. Zool..

[12] Smith S.M., Vale W.W. (2006). The role of the hypothalamic-pituitary-adrenal axis in neuroendocrine responses to stress. Dialogues Clin. Neurosci..

[13] Carroll J.A., Burdick Sanchez N.C. (2014). Overlapping physiological responses and endocrine biomarkers that are indicative of stress responsiveness and immune function in beef cattle. J. Anim. Sci..

[14] Vitku J., Kolatorova L., Hampl R. (2017). Occurrence and reproductive roles of hormones in seminal plasma. Basic Clin. Androl..

[15] Thun R., Eggenberger E., Zerobin K., Lüscher T., Vetter W. (1981). Twenty-four-hour secretory pattern of cortisol in the bull: Evidence of episodic secretion and circadian rhythm. Endocrinology.

[16] Blum J.W., Jans F., Moses W., Fröhli D., Zemp M., Wanner M., Hart I.C., Thun R., Keller U. (1985). Twenty-four-hour pattern of blood hormone and metabolite concentrations in high-yielding dairy cows: Effects of feeding low or high amounts of starch, or crystalline fat. Zentralbl. Veterinarmed. A.

[17] Pascual-Le Tallec L., Lombès M. (2005). The Mineralocorticoid Receptor: A Journey Exploring Its Diversity and Specificity of Action. Mol. Endocrinol..

[18] Smoak K.A., Cidlowski J.A. (2004). Mechanisms of glucocorticoid receptor signaling during inflammation. Mech. Ageing Dev..

[19] Heim C., Ehlert U., Hellhammer D.H. (2000). The potential role of hypocortisolism in the pathophysiology of stress-related bodily disorders. Psychoneuroendocrinology.

[20] Spencer R., Kalman B., Dhabhar F. (2011). Role of Endogenous Glucocorticoids in Immune System Function: Regulation and Counterregulation.

[21] Romero L.M., Dickens M.J., Cyr N.E. (2009). The reactive scope model—A new model integrating homeostasis, allostasis, and stress. Horm. Behav..

[22] Fernandez-Novo A., Pérez-Garnelo S.S., Villagrá A., Pérez-Villalobos N., Astiz S. (2020). The Effect of Stress on Reproduction and Reproductive Technologies in Beef Cattle—A Review. Animals.

[23] Kovács L., Jurkovich V., Bakony M., Szenci O., Póti P., Tőzsér J. (2014). Welfare implication of measuring heart rate and heart rate variability in dairy cattle: Literature review and conclusions for future research. Animal.

[24] Chu B., Marwaha K., Sanvictores T., Ayers D. (2022). Physiology, Stress Reaction.

[25] McCorry L.K. (2007). Physiology of the autonomic nervous system. Am. J. Pharm. Educ..

[26] von Borell E., Langbein J., Després G., Hansen S., Leterrier C., Marchant-Forde J., Marchant-Forde R., Minero M., Mohr E., Prunier A. (2007). Heart rate variability as a measure of autonomic regulation of cardiac activity for assessing stress and welfare in farm animals—A review. Physiol. Behav..

[27] Shaffer F., Ginsberg J.P. (2017). An Overview of Heart Rate Variability Metrics and Norms. Front. Public Health.

[28] Collier R.J., Renquist B.J., Xiao Y. (2017). A 100-Year Review: Stress physiology including heat stress. J. Dairy Sci..

[29] Bruckmaier R.M. (2005). Normal and disturbed milk ejection in dairy cows. Domest. Anim. Endocrinol..

[30] Buonaiuto G., Lopez-Villalobos N., Costa A., Niero G., Degano L., Mammi L.M.E., Cavallini D., Palmonari A., Formigoni A., Visentin G. (2023). Stayability in Simmental cattle as affected by muscularity and body condition score between calvings. Front. Vet. Sci..

[31] Nannoni E., Buonaiuto G., Martelli G., Lizzi G., Trevisani G., Garavini G., Sardi L. (2023). Influence of Increased Freedom of Movement on Welfare and Egg Laying Pattern of Hens Kept in Aviaries. Animals.

[32] Fazio F., Ferrantelli V., Cicero A., Casella S., Piccione G. (2015). Utility of Acute Phase Proteins as Biomarkers of Transport Stress in Ewes and Beef Cattle. Ital. J. Food Saf..

[33] Grandin T. (2010). Auditing animal welfare at slaughter plants. Meat Sci..

[34] Szenci O., Karen A., Bajcsy A.C., Gáspárdy A., de Sousa N.M., Beckers J.F. (2011). Effect of restraint stress on plasma concentrations of cortisol, progesterone and pregnancy associated-glycoprotein-1 in pregnant heifers during late embryonic development. Theriogenology.

[35] ESVE (2019). ESVE Veterinary Endocrinology External Quality Assessment Scheme. https://www.esve.org/esve/eve-qas/default.aspx.

[36] Hopster H., van der Werf J.T.N., Erkens J.H.F., Blokhuis H.J. (1999). Effects of repeated jugular puncture on plasma cortisol concentrations in loose-housed dairy cows. J. Anim. Sci..

[37] Giese H., Dilly M., Gundelach Y., Hoffmann G., Schmicke M. (2018). Influence of transrectal palpation training on cortisol levels and heart rate variability in cows. Theriogenology.

[38] European Society of Cardiology (1996). Heart rate variability: Standards of measurement, physiological interpretation and clinical use. Task Force of the European Society of Cardiology and the North American Society of Pacing and Electrophysiology. Circulation.

[39] Hagen K., Langbein J., Schmied C., Lexer D., Waiblinger S. (2005). Heart rate variability in dairy cows—Influences of breed and milking system. Physiol. Behav..

[40] Kovács L., Kézér F.L., Póti P., Jurkovich V., Szenci O., Nagy K. (2019). Short communication: Heart rate variability, step, and rumination behavior of dairy cows milked in a rotary milking system. J. Dairy Sci..

[41] Mohr E., Langbein J., Nürnberg G. (2002). Heart rate variability: A noninvasive approach to measure stress in calves and cows. Physiol. Behav..

[42] R Core Team (2022). A Language and Environment for Statistical Computing.

[43] Kuznetsova A., Brockhoff P.B., Christensen R.H.B. (2017). lmerTest Package: Tests in Linear Mixed Effects Models. J. Stat. Softw..

[44] Wickham H. (2016). ggplot2: Elegant Graphics for Data Analysis.

[45] Lay D.C., Friend T.H., Randel R.D., Jenkins O.C., Neuendorff D.A., Kapp G.M., Bushong D.M. (1996). Adrenocorticotropic hormone dose response and some physiological effects of transportation on pregnant Brahman cattle. J. Anim. Sci..

[46] Kovács L., Tőzsér J., Szenci O., Póti P., Kézér F.L., Ruff F., Gábriel-Tőzsér G., Hoffmann D., Bakony M., Jurkovich V. (2014). Cardiac responses to palpation per rectum in lactating and nonlactating dairy cows. J. Dairy Sci..

[47] Veissier I., Le Neindre P. (1988). Cortisol responses to physical and pharmacological stimuli in heifers. Reprod. Nutr. Dev. (1980).

[48] Chen Y., Stookey J., Arsenault R., Scruten E., Griebel P., Napper S. (2016). Investigation of the physiological, behavioral, and biochemical responses of cattle to restraint stress1. J. Anim. Sci..

[49] Hopster H., Bruckmaier R.M., Van der Werf J.T.N., Korte S.M., Macuhova J., Korte-Bouws G., van Reenen C.G. (2002). Stress Responses during Milking; Comparing Conventional and Automatic Milking in Primiparous Dairy Cows. J. Dairy Sci..

[50] von Keyserlingk M.A.G., Olenick D., Weary D.M. (2008). Acute Behavioral Effects of Regrouping Dairy Cows. J. Dairy Sci..

[51] Veronesi M.C., Tosi U., Villani M., Govoni N., Faustini M., Kindahl H., Madej A., Carluccio A. (2010). Oxytocin, vasopressin, prostaglandin F2α, luteinizing hormone, testosterone, estrone sulfate, and cortisol plasma concentrations after sexual stimulation in stallions. Theriogenology.

[52] Colborn D.R., Thompson D.L., Roth T.L., Capehart J.S., White K.L. (1991). Responses of cortisol and prolactin to sexual excitement and stress in stallions and geldings. J. Anim. Sci..

[53] Borg K.E., Esbenshade K.L., Johnson B.H. (1991). Cortisol, growth hormone, and testosterone concentrations during mating behavior in the bull and boar1. J. Anim. Sci..

[54] Pesenhofer G., Palme R., Pesenhofer R.M., Kofler J. (2006). Comparison of two methods of fixation during functional claw trimming-Walk-in crush versus tilt table-In dairy cows using faecal cortisol metabolite concentrations and daily milk yield as parameters. Wien. Tierarztl. Monatsschr..

[55] Heinrich M., Muller H., Fieseler H., Steiner A., Gottschalk J., Einspanier A., Spilke J., Mielenz N., Palme R., Baumgartner W. (2020). Cortisol concentration before, during and after sham foot trimming in German Holstein cows-the suitability of different matrices. Tierarztl. Prax. Ausg. G Grosstiere Nutztiere.

[56] Herskin M.S., Munksgaard L., Andersen J.B. (2007). Effects of social isolation and restraint on adrenocortical responses and hypoalgesia in loose-housed dairy cows1. J. Anim. Sci..

[57] Alam M.G., Dobson H. (1986). Effect of various veterinary procedures on plasma concentrations of cortisol, luteinising hormone and prostaglandin F2 alpha metabolite in the cow. Vet. Rec..

[58] Nakao T., Sato T., Moriyoshi M., Kawata K. (1994). Plasma cortisol response in dairy cows to vaginoscopy, genital palpation per rectum and artificial insemination. J. Vet. Med. Ser. A.

[59] Schönbom H., Kassens A., Hopster-Iversen C., Klewitz J., Piechotta M., Martinsson G., Kißler A., Burger D., Sieme H. (2015). Influence of transrectal and transabdominal ultrasound examination on salivary cortisol, heart rate, and heart rate variability in mares. Theriogenology.

