# Supplementary material for: Comparative Assessment of the Stress Response of Cattle to Common Dairy Management Practices
Source: Animals (Basel). 2023 Jun 26;13(13):2115. doi: 10.3390/ani13132115 (PMC10340065; doi:10.3390/ani13132115)
Supplement: Supplementary file 1 [file animals-13-02115-s001.zip › Supplementary Materials_ComparativeAssessStress.pdf]

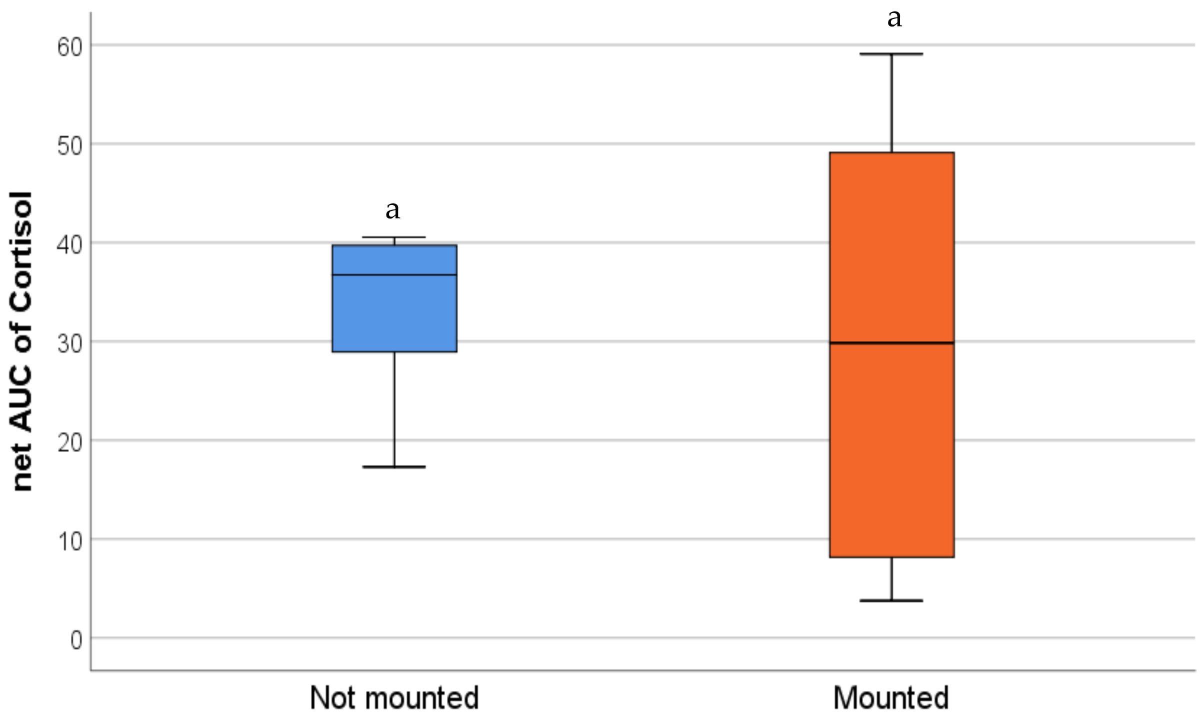

Figure S1. Stimulus, natural breeding, grouped by animals ‘Mounted’ and ‘Not mounted’ by the bull: No significant difference (*p* = 0.795) was found between cows experiencing social contact with a breeding bull (Not mounted: n=9) and cows that mated (Mounted: n=5) with a breeding bull.

Table S1. Descriptive statistics of bovine serum cortisol concentrations arranged by stimulus and subdivided by time point and daily status group

Stimulus	Status	Animals	Basal1	Basal2	Stimulus	Recovery1	Recovery2
			-40 minutes	-20 minutes	0 minutes	+20 minutes	+40 minutes
			nmol/l	nmol/l	nmol/l	nmol/l	nmol/l
		n	Mean ± SD	Mean ± SD	Mean ± SD	Mean ± SD	Mean ± SD
**AI**	Total	23	17.0 ± 4.9	28.0 ± 11.3	26.4 ± 11.3	20.6 ± 6.0	27.1 ± 11.0
	Co	15	16.5 ± 5.1	31.5 ± 12.1	24.6 ± 9.9	20.6 ± 5.7	26.8 ± 11.8
	Tr	8	18.0 ± 4.9	21.7 ± 6.5	29.5 ± 13.4	20.7 ± 6.9	27.6 ± 10.1
**ET**	Total	24	13.0 ± 3.7	17.1 ± 5.2	25.3 ± 13.0	16.3 ± 4.6	26.4 ± 9.8
	Co	16	11.7 ± 1.2	16.8 ± 3.6	19.3 ± 8.3	16.7 ± 5.0	32.1 ± 6.5
	Tr	8	15.2 ± 5.6	17.5 ± 7.5	35.8 ± 13.3	15.6 ± 3.9	15.6 ± 3.8
**MM**	Total	23	14.6 ± 3.8	18.1 ± 7.2	27.8 ± 17.0	21.0 ± 7.1	27.0 ± 10.1
	Co	15	14.2 ± 3.4	19.3 ± 7.4	18.2 ± 4.2	20.8 ± 7.3	28.1 ± 8.2
	Tr	8	15.3 ± 4.6	15.9 ± 6.6	44.5 ± 18.2	21.3 ± 6.7	25.1 ± 13.3
**VE**	Total	24	20.0 ± 7.3	27.7 ± 10.8	30.9 ± 12.6	28.8 ± 11.8	26.4 ± 8.7
	Co	16	20.1 ± 7.7	28.4 ± 11.5	31.0 ± 11.0	29.1 ± 11.5	26.2 ± 9.1
	Tr	8	20.0 ± 6.9	26.4 ± 9.9	30.7 ± 15.8	28.2 ± 13.2	26.8 ± 8.6
**EM**	Total	24	16.8 ± 4.1	17.5 ± 5.5	31.8 ± 12.7	16.5 ± 4.7	20.0 ± 5.9
	Co	12	15.9 ± 2.8	16.9 ± 5.4	30.1 ± 15.6	17.3 ± 5.6	22.1 ± 6.4
	Tr	12	17.7 ± 4.9	18.1 ± 5.7	33.4 ± 9.8	15.8 ± 3.9	18.0 ± 4.9
**US**	Total	23	15.3 ± 1.4	21.8 ± 9.2	28.2 ± 14.3	21.8 ± 10.3	19.5 ± 8.5
	Co	14	15.5 ± 1.5	21.2 ± 8.5	29.2 ± 13.4	18.5 ± 6.6	18.3 ± 4.9
	Tr	9	15.2 ± 1.4	22.7 ± 10.6	26.7 ± 16.2	27.5 ± 13.3	21.4 ± 12.4
**HT**	Total	20	12.1 ± 1.6	21.0 ± 12.0	40.5 ± 34.6	42.6 ± 36.7	35.1 ± 21.6
	Co	12	11.7 ± 1.4	20.8 ± 13.2	22.5 ± 8.6	18.1 ± 7.5	25.8 ± 10.5
	Tr	8	12.7 ± 1.9	21.2 ± 10.6	69.7 ± 41.5	79.3 ± 31.6	50.3 ± 26.8
**NB**	Total	14	27.4 ± 22.6	29.3 ± 13.0	71.7 ± 38.3	59.1 ± 31.4	53.7 ± 33.6
	NM	9	21.4 ± 18.7	26.4 ± 13.0	57.7 ± 21.3	57.6 ± 37.4	56.3 ± 39.9
	M	5	36.9 ± 27.2	34.5 ± 12.9	96.9 ± 51.2	61.8 ± 20.1	49.2 ± 21.2

Co (Control), Tr (Treatment), NM (not mounted), M (mounted), AI (artificial insemination), ET (embryo transfer), MM (morning milking), VE (veterinary examination), EM (evening milking), US (ultrasound examination), HT (hoof trimming), NB (natural breeding)

Table S2. Estimated mean bovine serum cortisol concentrations arranged by stimulus and subdivided into Control- and Treatment-groups

**Stimulus**	**Status**	**Animals**	**Basal1**	**Basal2**	**Stimulus**	**Recovery1**	**Recovery2**
			-40 minutes	-20 minutes	0 minutes	+20 minutes	+40 minutes
			nmol/l	nmol/l	nmol/l	nmol/l	nmol/l
		n	Estimated mean [95% CI]	Estimated mean [95% CI]	Estimated mean [95% CI]	Estimated mean [95% CI]	Estimated mean [95% CI]
**AI**	Co	15	15.8 [12.9, 19.2]	29.1 [23.9, 35.5]	22.7 [18.6, 27.7]	19.8 [16.1, 24.3]	24.5 [20.0, 29.8]
	Tr	8	17.9 [13.8, 23.3]	21.5 [16.5, 27.9]	27.9 [21.4, 36.2]	20.2 [15.6, 26.3]	26.5 [20.0, 35.0]
**ET**	Co	16	11.7 [9.6,14.1]	16.4 [13.4, 20.0]	17.8[a] [14.6, 21.7]	16.0 [13.1, 19.5]	31.3[a] [25.4, 38.4]
	Tr	8	14.7 [11.3, 19.2]	16.6 [12.7, 21.5]	34.1[b] [26.2, 44.3]	15.5 [11.9, 20.1]	15.4[b] [11.6, 20.4]
**MM**	Co	15	14.1 [11.6, 17.2]	18.4 [15.2, 22.3]	18.2[a] [14.9, 22.2]	20.2 [16.6, 24.4]	27.3 [22.6, 33.1]
	Tr	8	14.5 [11.2, 18.9]	14.7 [11.3, 19.1]	40.0[b] [30.8, 52.0]	20.1 [15.4, 26.1]	22.1 [17.0, 28.7]
**VE**	Co	16	19.1 [15.7, 23.1]	26.5 [21.8, 32.4]	29.4 [24.1, 35.8]	27.6 [22.8, 33.4]	25.2 [20.7, 30.8]
	Tr	8	19.0 [14.6, 24.7]	24.7 [19.0, 32.1]	27.5 [21.1, 35.7]	26.2 [20.1, 34.0]	25.6 [19.7, 33.3]
**EM**	Co	12	16.0 [12.8, 20.0]	16.6 [13.3, 20.8]	26.9 [21.5, 33.6]	17.0 [13.6, 21.3]	21.7 [17.3, 27,1]
	Tr	12	16.9 [13.7, 21.0]	17.2 [13.9, 21.3]	31.8 [25.7, 39.4]	15.3 [12.4, 19.0]	17.2 [13.9, 21.4]
**US**	Co	14	15.8 [12.6, 19.8]	20.2 [16.5, 24.9]	27.1 [22.2, 33.0]	18.0 [14.7, 21.9]	18.2 [14.9, 22.2]
	Tr	9	15.0 [11.7, 19.1]	20.5 [16.0, 26.2]	23.1 [18.1, 29.6]	24.5 [18.9, 31.9]	18.8 [14.7, 24.1]
**HT**	Co	12	11.8 [9.5, 14.6]	18.2 [14.9, 22.4]	21.5[a] [17.5, 26.4]	17.3[a] [14.0, 21.4]	24.5[a] [19.9, 30.0]
	Tr	8	12.5 [9.6, 16.2]	18.9 [14.6, 24.6]	57.2[b] [44.0, 74.4]	72.9[b] [56.1, 94.8]	44.0[b] [33.8, 57.2]
**NB**	NM	9	17.9[a] [13.8, 23.3]	24.0 [18.7, 30.7]	59.9 [47.8, 74.9]	49.4 [38.6, 63.3]	45.9 [35.8, 58.8]
	M	5	29.6[b] [21.3, 41.2]	32.4 [23.3,45.1]	60.7 [39.6, 92.9]	58.5 [42.0, 81.5]	43.5 [31.2, 60.5]

Co (Control), Tr (Treatment), NM (not mounted), M (mounted), AI (artificial insemination), ET (embryo transfer), MM (morning milking), VE (veterinary examination), EM (evening milking), US (ultrasound examination), HT (hoof trimming), NB (natural breeding), Sig. diff. between Control- and Treatment-groups $^{ab}p < 0.05$.

Table S3. Estimated mean values of heart rate variability parameters, subdivided by stimulus

Stimulus	Parameter	Pre-stimulus	Stimulus	Post-stimulus
		Estimated mean [95% CI]	Estimated mean [95% CI]	Estimated mean [95% CI]
**Artificial insemination**	Mean HR (bpm)	84 [78, 90]	84 [77, 91]	81 [75, 87]
	Mean RR (ms)	721 [675, 768]	719 [667, 772]	743 [697, 790]
	RMSSD (ms)	5.3 [3.7, 7.5]	5.7 [3.9, 8.4]	6.3 [4.5, 9.0]
(n=4)	HF band (nu)	5.6 [3.9, 8.1][a]	6.3 [3.9, 9.9][a,b]	9.1 [6.3, 13.2][b]
	SD2/SD1 Ratio	4.8 [4.0, 5.6][a]	4.8 [3.8, 5.8][a,b]	4.0 [3.2, 4.8][b]
**Embryo transfer**	Mean HR (bpm)	93 [87, 99]	93 [86, 99]	93 [87, 99]
	Mean RR (ms)	639 [592, 686]	639 [587, 691]	641 [594, 688]
	RMSSD (ms)	3.1 [2.1, 4.4]	2.9 [2.0, 4.3]	2.9 [2.0, 4.2]
(n=5)	HF band (nu)	11.7 [8.2, 16.7]	9.0 [5.8, 13.9]	9.7 [6.8, 13.9]
	SD2/SD1 Ratio	4.3 [3.5, 5.2]	4.6 [3.6, 5.5]	4.9 [4.1, 5.7]
**Morning milking**	Mean HR (bpm)	85 [78, 91]	87 [80, 94]	85 [79, 91]
	Mean RR (ms)	733 [685, 780]	719 [665, 772]	727 [679, 774]
	RMSSD (ms)	9.1 [6.3, 12.9]	8.8 [5.9, 12.9]	7.4 [5.2, 10.6]
(n=4)	HF band (nu)	13.0 [8.9, 19.0][a]	6.4 [4.0, 10.2][b]	7.3 [5.0, 10.7][b]
	SD2/SD1 Ratio	3.9 [3.1, 4.7]	4.7 [3.7, 5.7]	4.5 [3.7, 5.3]
**Veterinary examination**	Mean HR (bpm)	87 [81, 93]	88 [81, 95]	84 [78, 90]
	Mean RR (ms)	692 [645, 739]	686 [635, 738]	713 [666, 760]
	RMSSD (ms)	6.8 [4.8, 9.7][a]	11.3 [7.5, 16.9][b]	6.1 [4.2, 8.7][a]
(n=5)	HF band (nu)	11.7 [8.2, 16.7]	16.4 [10.6, 25.4]	11.0 [7.7, 15.7]
	SD2/SD1 Ratio	3.8 [3.0, 4.6]	4.1 [3.2, 5.0]	3.6 [2.8, 4.4]
**Evening milking**	Mean HR (bpm)	93 [86, 100]	92 [84, 101]	89 [82, 97]
	Mean RR (ms)	655 [600, 711]	662 [600, 724]	682 [626, 738]
	RMSSD (ms)	5.2 [3.5, 7.8]	4.6 [3.0, 7.2]	4.6 [3.0, 6.8]
(n=3)	HF band (nu)	3.6 [2.3, 5.8][a]	6.2 [3.5, 10.8][a,b]	6.2 [3.9, 9.8][b]
	SD2/SD1 Ratio	5.4 [4.4, 6.4]	4.6 [3.5, 5.8]	4.6 [3.6, 5.6]
**Ultrasound examination**	Mean HR (bpm)	105 [92, 119][a]	100 [86, 114][a,b]	97 [84, 111][b]
	Mean RR (ms)	577 [468, 685]	607 [493, 721]	619 [511, 728]
	RMSSD (ms)	3.5 [1.5, 8.3]	3.0 [1.3, 7.4]	2.6 [1.1, 6.1]
(n=2)	HF band (nu)	9.5 [5.0, 18.1]	7.3 [3.4, 15.5]	11.5 [6.0, 22.0]
	SD2/SD1 Ratio	5.2 [3.5, 6.8]	4.8 [3.0, 6.6]	5.1 [3.4, 6.7]
**Hoof trimming**	Mean HR (bpm)	95 [88, 101]	95 [88, 103]	99 [87, 110]*
	Mean RR (ms)	651 [601, 701]	654 [597, 711]	640 [557, 724]*
	RMSSD (ms)	6.9 [4.8, 10.0][a]	7.2 [4.8, 10.9][a,b]	11.6 [6.6, 20.6]*[b]
(n=3)	HF band (nu)	6.8 [4.5, 10.2]	4.1 [2.4, 7.0]	3.8 [1.6, 8.9]*
	SD2/SD1 Ratio	4.7 [3.8, 5.6]	4.8 [3.7, 5.8]	5.5 [3.8, 7.2]*

*Measurements from only one cow available during Post-stimulus Phase of the hoof trimming experiment (n=1). Significant differences between experimental Phases ($^{ab}p < 0.05$).
